# Biofidelic dynamic compression of human cortical spheroids reproduces neurotrauma phenotypes

**DOI:** 10.1242/dmm.048916

**Published:** 2021-12-22

**Authors:** Aaron R. Shoemaker, Ian E. Jones, Kira D. Jeffris, Gina Gabrielli, Alyssa G. Togliatti, Rajeswari Pichika, Eric Martin, Evangelos Kiskinis, Colin K. Franz, John D. Finan

**Affiliations:** 1 Department of Neurosurgery, NorthShore University Health System, Evanston, IL 60201, USA; 2 Department of Mechanical and Industrial Engineering, University of Illinois at Chicago, Chicago, IL 60607, USA; 3 Shirley Ryan AbilityLab, Chicago, IL 60611, USA; 4 Department of Physical Medicine and Rehabilitation, Northwestern University Feinberg School of Medicine, Chicago, IL 60611, USA; 5 The Ken & Ruth Davee Department of Neurology, Northwestern University Feinberg School of Medicine, Chicago, IL 60611, USA

**Keywords:** Traumatic brain injury, Human, *In vitro*, Spheroid

## Abstract

Fundamental questions about patient heterogeneity and human-specific pathophysiology currently obstruct progress towards a therapy for traumatic brain injury (TBI). Human *in vitro* models have the potential to address these questions. Three-dimensional spheroidal cell culture protocols for human-origin neural cells have several important advantages over their two-dimensional monolayer counterparts. Three-dimensional spheroidal cultures may mature more quickly, develop more biofidelic electrophysiological activity and/or reproduce some aspects of brain architecture. Here, we present the first human *in vitro* model of non-penetrating TBI employing three-dimensional spheroidal cultures. We used a custom-built device to traumatize these spheroids in a quantifiable, repeatable and biofidelic manner, and correlated the heterogeneous mechanical strain field with the injury phenotype. Trauma reduced cell viability, mitochondrial membrane potential and spontaneous synchronous electrophysiological activity in the spheroids. Electrophysiological deficits emerged at lower injury severities than changes in cell viability. Also, traumatized spheroids secreted lactate dehydrogenase, a marker of cell damage, and neurofilament light chain, a promising clinical biomarker of neurotrauma. These results demonstrate that three-dimensional human *in vitro* models can reproduce important phenotypes of neurotrauma *in vitro*.

## Abstract (precis)

**Summary:** Self-organizing spheroids of astrocytes and neurons derived from human induced pluripotent stem cells reproduce phenotypes associated with traumatic brain injury when subjected to dynamic compression.

## INTRODUCTION

Traumatic brain injury (TBI) is diagnosed in ∼2.5 million patients every year in the United States. Most TBI is mild but 282,000 of these patients are hospitalized annually (Taylor et al., 2017), indicating moderate to severe injury. TBI affects individuals at every stage of life but it is most common among children aged 0-4 years, so it poses an enormous pediatric healthcare challenge (Cancelliere et al., 2017). Even mild TBI increases the risk of neurodegenerative disorders, such as Parkinson's (Gardner et al., 2015) and Alzheimer's disease (Barnes et al., 2018). TBI can be divided into penetrating TBI and closed TBI, which is more common. In closed TBI, head impact violently accelerates the head. The human brain is soft and heavy so it shifts and stretches under its own weight when the head accelerates (Alshareef et al., 2018). This deformation triggers a complex multimodal pathology, including but not limited to excitotoxicity (Palmer et al., 1993), inflammation (Johnson et al., 2013), mitochondrial failure (Cheng et al., 2012), axonal transport failure (Tang-Schomer et al., 2012), cytoskeletal degradation (Buki et al., 1999), oxidative stress (Tyurin et al., 2000) and membrane permeabilization (Pettus et al., 1994). These pathologies can be reproduced using agents ranging from cytokines to toxins to detergents but it is not trivial to reproduce them simultaneously with the right intensity and time course to reproduce clinical TBI. An attractive alternative is to apply biofidelic deformation to a biofidelic surrogate for the human brain and let these pathologies emerge spontaneously.

The human brain is particularly vulnerable to inertial loading relative to other species because it is unusually massive (Panzer et al., 2014). In animals such as macaques and pigs, accelerations must be scaled up to compensate for the smaller brain mass so the brain will inertially deform to the point of injury (Ommaya et al., 1967). The scaled accelerations required to deform the rodent brain through inertial loading are enormous and difficult to apply experimentally (Davidsson and Risling, 2011). Therefore, most preclinical TBI models load a stationary human brain surrogate via a solid or liquid interface instead of attempting to deform it with acceleration (Dixon et al., 1987; Dixon et al., 1991; Saikumar et al., 2020). In animal models, loading deforms brain cells and ruptures the blood brain barrier simultaneously. However, *in vitro* models can address questions about the brain parenchyma independently from the vasculature. Various surrogates for the brain have been used in *in vitro* models, including two-dimensional monolayers (Ellis et al., 1995; Phillips et al., 2019), three-dimensional hydrogel cultures (Bar-Kochba et al., 2016; LaPlaca et al., 2005; Tang-Schomer et al., 2014) and organotypic slices (Morrison et al., 2006). Two-dimensional systems are typically loaded by stretching an extensible culture substrate (Morrison et al., 2011), whereas three-dimensional hydrogels are compressed (Bar-Kochba et al., 2016; Tang-Schomer et al., 2014) or sheared (Cullen et al., 2007). Recently, human induced pluripotent stem cells (hiPSCs) have increased the power of *in vitro* models of neurological disease (Hargus et al., 2014). These cells can be generated in large numbers to support high-content screens (Drawnel et al., 2014). Their genome is patient specific and editable (Barral and Kurian, 2016). This feature allows unique studies of genetic factors, which are important in TBI (Finan et al., 2018). However, to date, hiPSCs have only been applied to model neurotrauma once in two-dimensional monolayer cultures (Sherman et al., 2016). Three-dimensional culture protocols have dramatically expanded the range of brain features and pathophysiology that can be reproduced *in vitro* (Amin and Pasca, 2018). Three-dimensional cultures are particularly attractive for TBI research. Brain tissue is compliant and incompressible so it expands horizontally when it is compressed vertically (Holburn, 1943). As a result, axial compression and planar tension can interact in a three-dimensional tissue in a way they cannot in a two-dimensional monolayer. Three-dimensional cultures can also reveal pathological changes in volume (Watanabe et al., 2017), which are among the most important clinical complications of TBI (Finan et al., 2016; Unterberg et al., 2004).

Organoid models create exciting opportunities for *in vitro* modeling of neurological disease. However, simulating TBI in spheroidal cultures poses some challenges. These cultures are difficult to deform at strain rates and magnitudes representative of clinical TBI. Also, spheroids differ from more convenient geometries, such as cylinders and cubes, because they generate spatially heterogeneous strain fields when compressed (Nguyen et al., 2010). Nevertheless, the most advanced culture protocols in the field spontaneously adopt spheroidal geometries (Lancaster et al., 2013; Pasca et al., 2015). Therefore, this study embraced the canonical spheroidal geometry of three-dimensional human *in vitro* cultures and adapted the experimental apparatus and biomechanical analysis to it. We designed the apparatus to apply strains at biofidelic rates, as well as biofidelic magnitudes, because rapidly applied strains are more injurious than gradually applied strains (Ahmadzadeh et al., 2014; Kang and Morrison, 2015). We traumatized human cortical spheroids in a 96-well format and they exhibited clinically relevant injury phenotypes, including changes in cell viability, mitochondrial membrane potential, electrophysiological activity and the release of clinically established biomarkers.

## RESULTS

### Compressive injury of human cortical spheroids

**Fig. 1.**
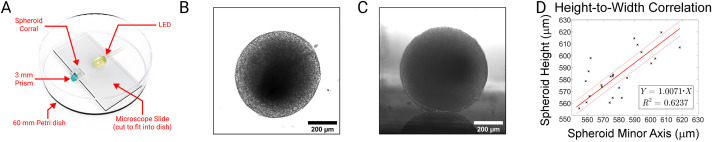
**Spheroid width accurately predicts spheroid height.** (A) Apparatus for imaging a spheroid on horizontal and vertical planes. (B) Bright-field image of spheroid taken from below. (C) Bright-field image of spheroid taken from the side. (D) Linear regression of spheroid height against spheroid minor axis when viewed from below (*n*=24, *P*<0.001).

We dynamically compressed cortical spheroids derived from hiPSCs between two rigid flat surfaces to injure them. The first step was accurately estimating the height of the spheroids. We assumed height was equal to the width (defined as the minor axis of the elliptical outline of the spheroid in a two-dimensional image). To validate this assumption, we imaged a subset of spheroids in the horizontal and vertical planes using a prism (Fig. 1A-C), and measured height and width directly. Average spheroid width was 578.1±17.6 µm (s.d.); average spheroid height was 582.3±19.9 µm (s.d.) (*n*=24). Therefore, these spheroids were almost spherical and their size was consistent within a single batch. Moreover, height and width were correlated within their narrow ranges with a slope of 1.0071 and R^2^=0.6237 (Fig. 1D, *P*<0.001) so width predicted height well. Spheroid size varied somewhat from batch to batch but was consistent within batches (s.d. of width <4% of average width, Table S1). Therefore, we measured average spheroid size for each batch shortly before each injury experiment and adjusted the initial position of the injury apparatus accordingly. Within each batch, we used spheroid-specific size to compute spheroid-specific strain metrics for a given compressive displacement.

**Fig. 2.**
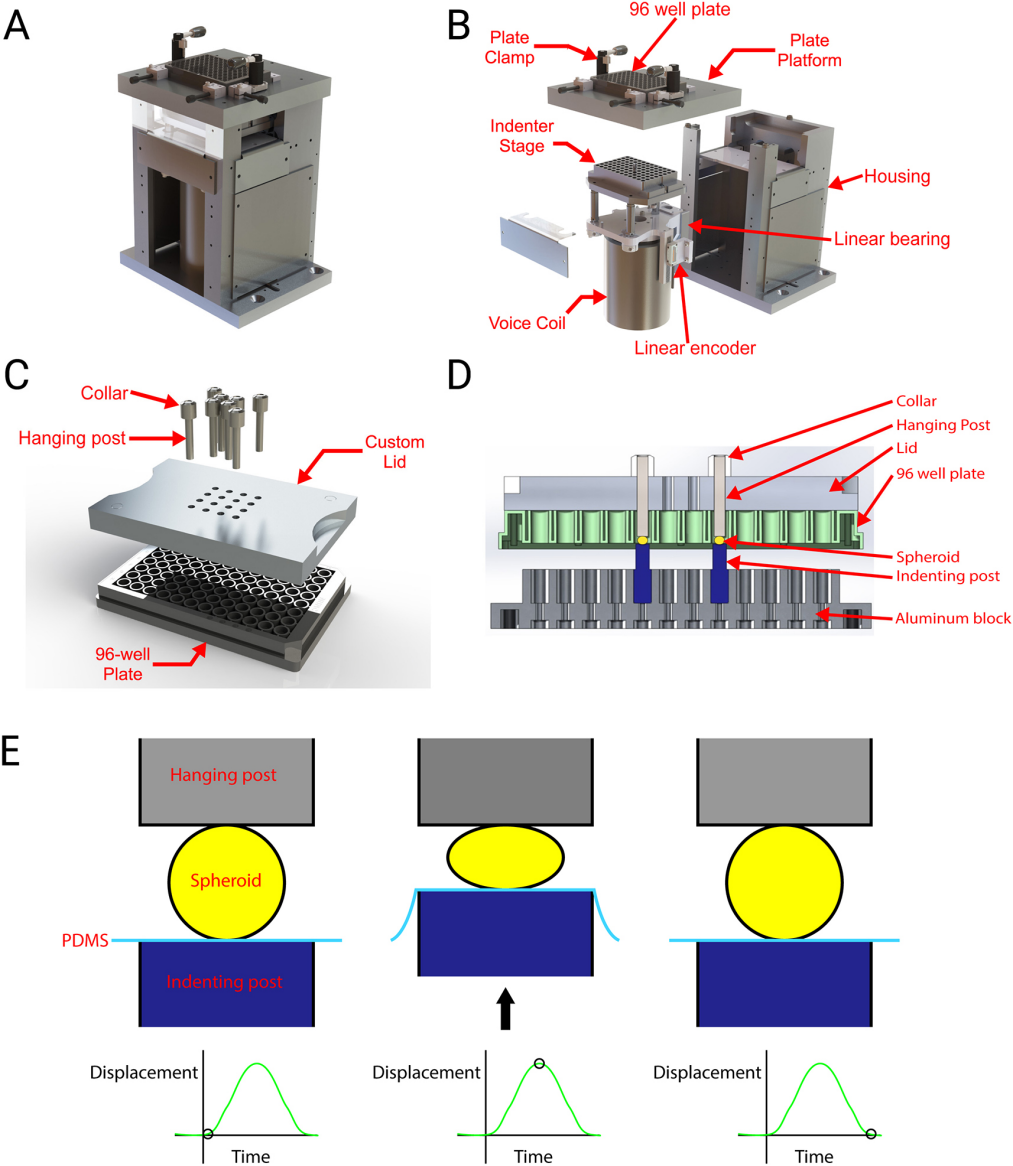
**Dynamic compressive injury of a human cortical spheroid.** (A) Custom-built injury device. (B) Exploded view of custom-built injury device. (C) Exploded view of lid, drop-in posts and 96-well plate. (D) Cross-sectional view of spheroids in injury apparatus at the initial position for the injury protocol. A PDMS membrane forms the bottom of the plate but is not shown in this image for clarity. (E) Schematic of the sequence of events during injury. At the start of the injury, the hanging post is touching the top of the spheroid and the indenting post is lightly pressing the PDMS membrane against the bottom of the membrane. The spheroid is therefore held between two effectively rigid surfaces but not compressed. Then, the voice coil drives the indenting post upwards to compress the spheroid. Then, the indenting post lowers to end the pulse of compression.

Once the size of the spheroids was established, we transferred them into stretchable 96-well plates [i.e. standard bottomless 96-well plates to which sheets of transparent polydimethylsiloxane (PDMS) had been adhered (Sherman et al., 2016; Phillips et al., 2018)] and traumatized them using a custom-built bench top instrument. The instrument drove a stage along a vertical axis towards an opening in a fixed platform above it. The stage supported an array of removable rigid cylindrical indenters aligned with the wells of a stretchable 96-well plate clamped to the platform (Fig. 2A,B). A custom-built lid sitting on the 96-well plate supported a series of drop-in posts (Fig. 2C,D). We adjusted the position of the posts above the spheroids and the indenters below so the space between them would match the average height of the spheroids at the start of the experiment. Then, the voice coil drove the stage rapidly up and down through a prescribed displacement to compress the spheroids (Fig. 2E). No cracking, shape change or other evidence of permanent damage was observed after compression. The variation in the dimensions of the posts and indenters were small relative to the size of the spheroidal cultures (Fig. S1). The duration of the displacement pulse was ∼30 ms, with almost no dependence on pulse amplitude (see Table S2). This duration was consistent with the duration of head impacts in human cadaveric heads (Zou et al., 2007).

### Mathematical modeling of the strain field

**Fig. 3.**
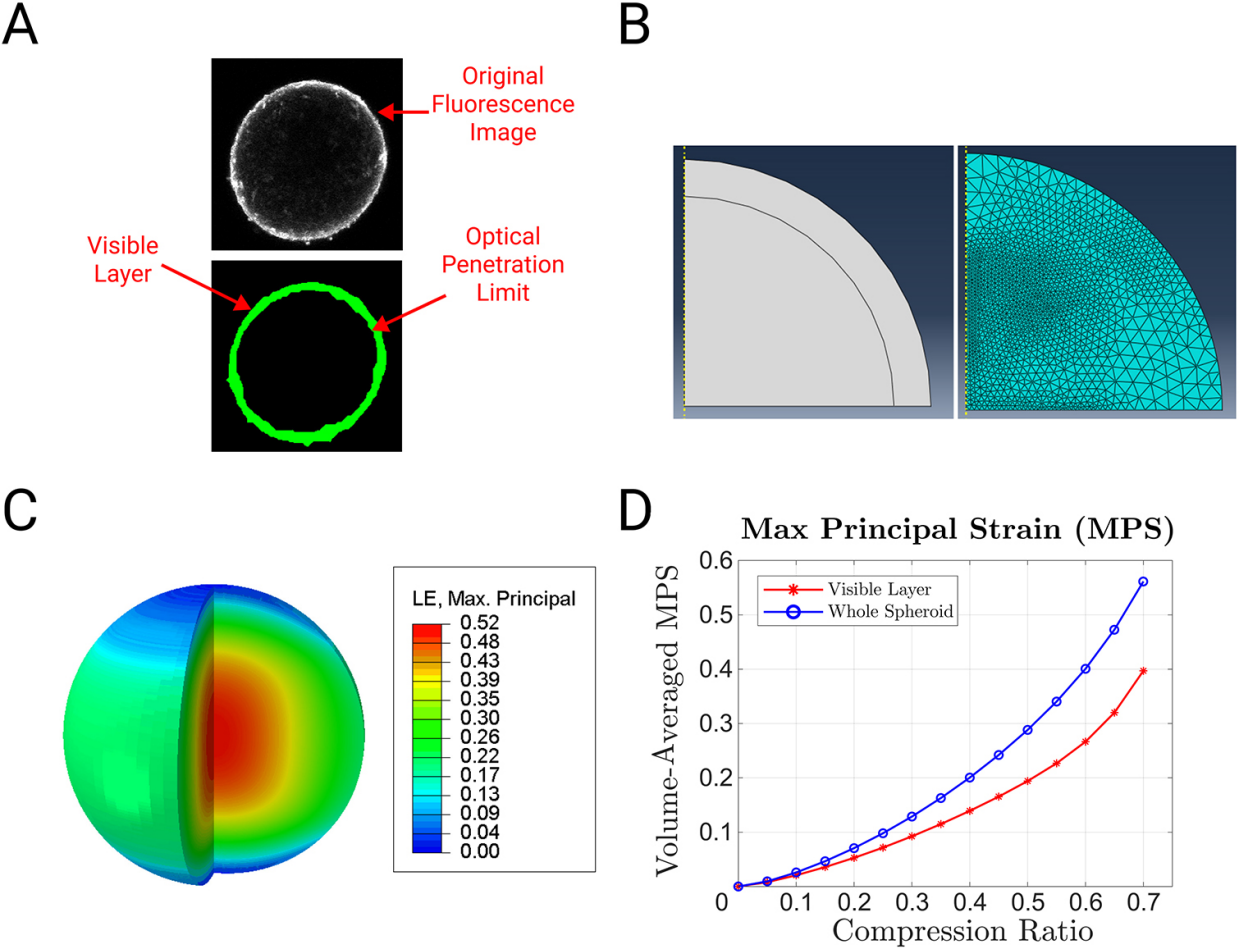
**Mathematical modeling of strain field in a compressed spheroid.** (A) Fluorescent image of a spheroid stained with Calcium 6 (top panel) and automated segmentation of the image (bottom panel) to determine the thickness of the visible region. (B) Finite element model geometry (left panel) and mesh (right panel). (C) Color map of MPS in spheroid at 50% compression, mapped on to the undeformed geometry. LE, logarithmic strain. (D) MPS_visible_ and MPS_whole_ for various compression ratios (solid lines represent the fourth order polynomial fit with R^2^>0.99).

We used finite element analysis (Voo et al., 1996) to deduce the strain field in the spheroid from the compression ratio (i.e. the amplitude of the injury pulse divided by the initial height). Unconfined compression of a sphere between flat rigid surfaces creates a time-varying mixed boundary condition. Inside the contact area, the position of the rigid surface defines a displacement boundary condition. Outside the contact area, the absence of traction on the surface defines a traction boundary condition. The contact area is small initially but it expands during compression. Single photon confocal microscopy could penetrate only 45 µm into the spheroid in our hands (Fig. 3A), so we partitioned this region of the mesh and considered it separately in some subsequent analyses (Fig. 3B). We used the maximum principal strain (MPS) as a scalar representation of the strain tensor (Gabler et al., 2018). MPS peaked near the core of spheroid far from the strain-relieving free surface. It assumed an intermediate value near the free surface and a minimal value near the contact surfaces, where the surface friction countered the tension due to Poisson effects (Lai et al., 1995; Fig. 3C; note that Poisson effects dominate the MPS term in a compressive loading mode because tension is positive and compression is negative). We labeled the average MPS in the visible region MPS_visible_ and the average MPS in the whole spheroid MPS_whole_. MPS_visible_ excluded the highest strain values in the strain field so it increased with compression ratio more slowly than MPS_whole_ (Fig. 3D).

### Trauma damages cells, depolarizes mitochondria and triggers release of NF-L

**Fig. 4.**
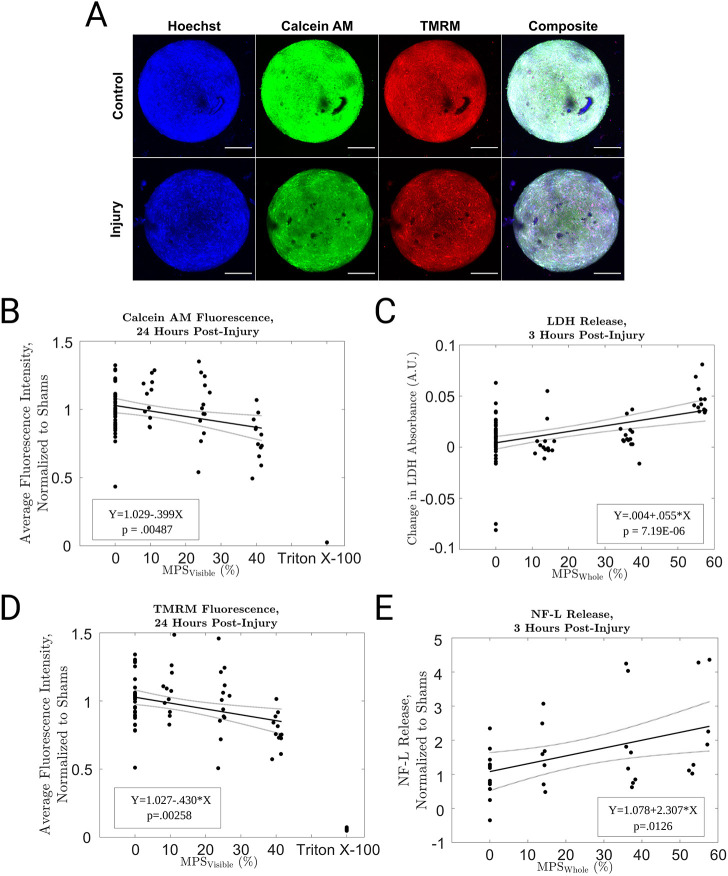
**Trauma impacts cell heath in spheroids and causes them to release injury biomarkers.** (A) Maximum intensity projections of confocal images of spheroids labeled with Hoechst 33342, Calcein AM and TMRM, before and after injury. Scale bars: 150 µm. (B) The effect of various levels of trauma (and Triton X-100 treatment) on Calcein AM fluorescent intensity. (C) The effect of various levels of trauma on LDH secretion. (D) The effect of various levels of trauma (and Triton X-100 treatment) on TMRM fluorescence intensity. (E) The effect of various levels of trauma on the secretion of NF-L. Linear regression along with confidence intervals and fit parameters are superimposed on panels B-E. A.U., arbitrary units.

We quantified Calcein AM fluorescence to measure the damage to cells 24 h after trauma (Fig. 4A). Calcein AM is a membrane permeable small molecule that specifically labels living cells (see Fig. S4 for visualization of staining distribution at cellular length scales). Calcein AM intensity fell steadily with increasing MPS_visible._ However, even the most severely strained spheroids had much brighter signal than the negative control group, which was treated with 0.5% Triton X-100 for 24 h (Fig. 4B) to completely destroy the plasma membrane and kill the cells, thereby reproducing the worst effects that could be expected from mechanical trauma. We confirmed the relationship between strain and damage using a lactate dehydrogenase (LDH) assay, which is not limited to the visible domain of the spheroid. LDH is an enzyme found in most cells that enters the medium when the cell has compromised membrane integrity. LDH concentrations rose with increasing MPS_whole_ (Fig. 4C).

Dysfunctional mitochondria play a dual role in trauma pathology because they produce reactive oxygen species that poison the cell and also fail to produce the energy needed to restore cellular homeostasis (Cheng et al., 2012; Ellis et al., 1995). Mitochondrial dysfunction after trauma occurs *in vitro* (Ellis et al., 1995), in small animals (Ren et al., 2020) and in human subjects (Aygok et al., 2008). We measured mitochondrial membrane potential using three-dimensional confocal microscopy and tetramethylrhodamine methyl ester perchlorate (TMRM) staining. TMRM is a cell-permeable positively charged small molecule stain that accumulates in healthy mitochondria. The signal declined steadily with increasing MPS_visible_. However, even the most severely injured spheroids had much brighter signal than the Triton X-100-treated negative control group (the purpose of the Triton X-100 treatment is to reduce the mitochondrial signal to the lowest possible level), indicating that many mitochondria remained viable after injury (Fig. 4D). In order to predict clinical success, preclinical TBI models must measure clinically relevant biomarkers (DeWitt et al., 2018). Neurofilament light chain (NF-L) concentration is a promising clinical TBI biomarker (Shahim et al., 2017). Therefore, we measured NF-L released into the medium after trauma using an ELISA assay. NF-L levels increased with increasing MPS_whole_, indicating that this model could be used to discover agents that mitigate NF-L release from injured human cortical cells (Fig. 4E).

### Trauma suppresses electrophysiological activity

**Fig. 5.**
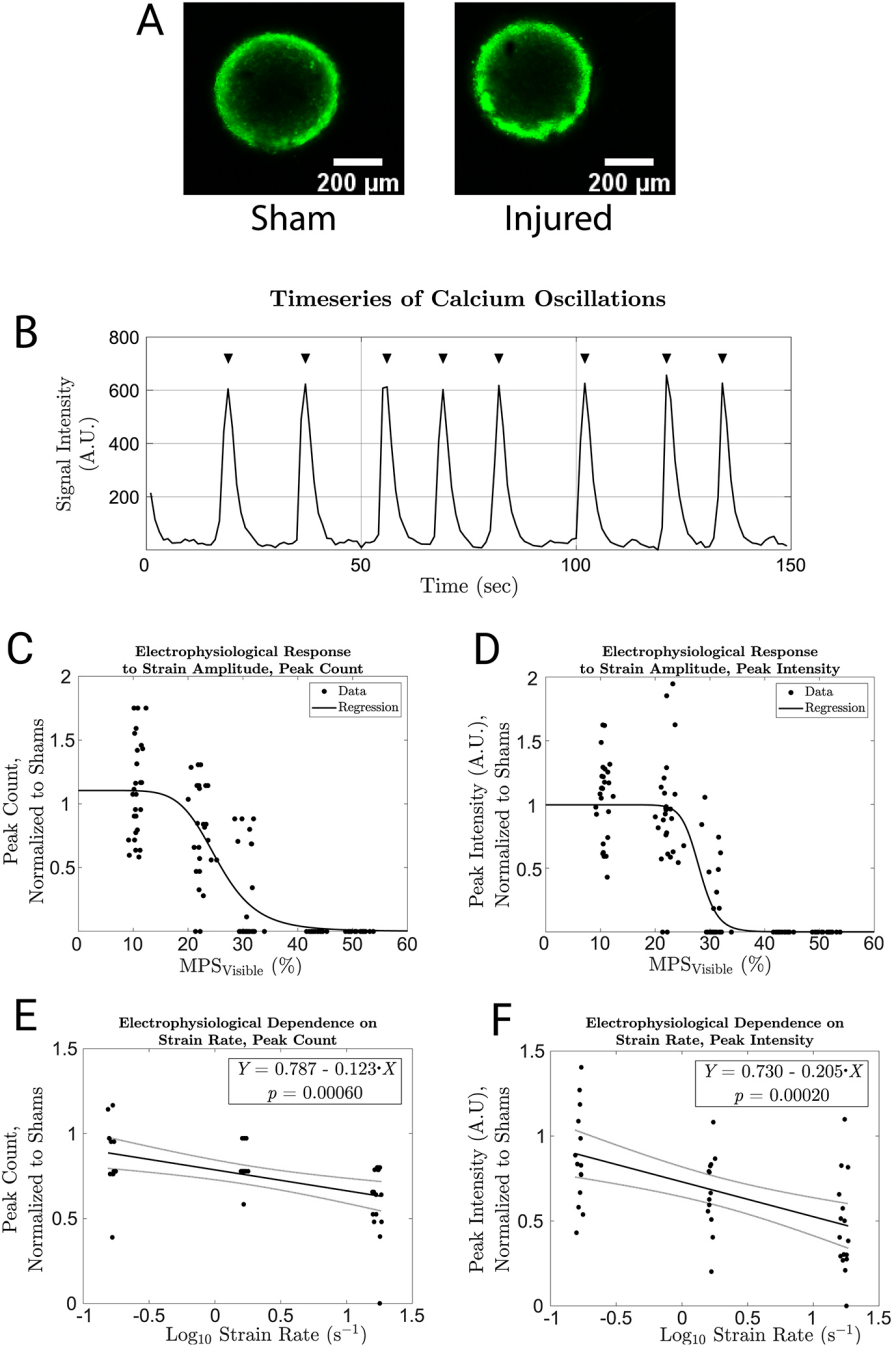
**Electrophysiological changes after trauma.** (A) Average intensity projections of time series confocal images of Calcium 6-stained sham and injured spheroids. (B) Typical time series of calcium oscillations from a healthy human cortical spheroid. Black triangles indicate automatically identified peaks in the series. (C) The effect of various levels of trauma on the number of detected peaks. The black line plots a sigmoidal regression (see Supplementary Materials and Methods and Table S3) of the data (R^2^=0.675). (D) The effect of various levels of trauma on the amplitude of the detected peaks. The black line plots a sigmoidal regression of the data (R^2^=0. 698). (E) The number of detected peaks after compression to MPS_visible_=0.3 at various strain rates. A linear regression is superimposed on the data along with confidence bounds and fit parameters (R^2^=0.229). (F) The amplitude of detected peaks after compression to MPS_visible_=0.3 at various strain rates. A linear regression is superimposed on the data along with confidence bounds and fit parameters (R^2^=0. 267). A.U., arbitrary units.

Electroencephalography (EEG) detects electrophysiological dysfunction in human subjects, and EEG parameters can serve as functional biomarkers of clinical TBI (Corbin-Berrigan et al., 2020). The spheroids used in this study displayed synchronous (see Fig. S5) spontaneous electrical activity, which we visualized with the calcium indicator Calcium 6 (see Fig. 5A) and quantified with time series fluorescent microscopy (Fig. 5B). The number and intensity of calcium waves decreased with increasing MPS_visible_ (Fig. 5C,D). They also decreased with increasing strain rate for a constant strain magnitude (MPS_visible_=0.3), indicating rate sensitivity (Fig. 5E,F). Electrophysiological activity was completely abolished for MPS_visible_ values at which Calcein AM and TMRM intensities were far above their negative control values. Therefore, electrophysiological dysfunction is unlikely to be a direct consequence of cell death.

## DISCUSSION

TBI is a major public health challenge that has motivated a significant but thus far unsuccessful effort to find an effective therapy. A total of 191 clinical trials (Bragge et al., 2016), including at least 33 large multicenter phase III trials, have been conducted for TBI but we remain without a US Food and Drug Administration (FDA)-approved treatment for this disorder (Maas et al., 2010). Human *in vitro* models of TBI have exciting potential to address three of the key reasons why clinical trials fail. First, human TBI pathology differs from TBI pathology in other species. Progesterone mitigated TBI pathology in more than 300 animal studies but failed at clinical trial (Stein, 2015). A human *in vitro* model of TBI introduces human pathophysiology at the preclinical stage. Second, current preclinical TBI models are low throughput, so generating candidates for clinical trials is slow and expensive. Human *in vitro* models enable high content screens because hiPSC-derived cultures can be generated in large numbers. Finally, the trajectory of clinical morbidity varies widely among TBI patients receiving identical therapy (Gardner et al., 2019). This variation overwhelms the effect of a therapeutic candidate at trial, rendering it statistically insignificant (Maas et al., 2010). The solution is to stratify the trial based on prognosis but genetic factors that determine prognosis are poorly understood. A human *in vitro* model can reveal an individual's genetically determined disease course without *a priori* understanding of the associated genetic determinants. Therefore, human *in vitro* models have the potential to solve important problems in preclinical TBI research (Franz et al., 2019). However, none of this potential can be realized without a biofidelic disease phenotype.

The first step to a biofidelic injury phenotype is a biofidelic mechanical insult. As discussed in the Introduction, it is difficult to reproduce the inertial stresses generated by acceleration of the massive human brain in a smaller experimentally convenient model system. The alternative is to match the strain amplitude in the experimental model to that of clinical TBI, and that is the approach taken in this study. Previously reported three-dimensional *in vitro* neurotrauma models used cylindrical or cuboid hydrogel cultures (LaPlaca et al., 2005; Bar-Kochba et al., 2016; Tang-Schomer et al., 2014). These shapes are convenient because they produce spatially homogeneous strain fields. However, when brain organoid cultures self-organize, they typically assume spheroidal shapes. Self-organization improves the biofidelity of the culture (Pasca et al., 2015; Lancaster et al., 2013). It permits high cell densities that accommodate paracrine and electrophysiological communication between cells. These benefits come at the price of biomechanical complexity. This study loaded spheroidal cultures in unconfined compression (Fig. 2E), which produced a heterogeneous strain field (Fig. 3C). There are several options for scalar parameters to summarize the strain tensor at any point in space. MPS (Bandak, 1995; Takhounts et al., 2013) is a popular choice in TBI biomechanics. MPS emphasizes tension over compression as tension is positive. Even when the macroscopic loading is compressive, tension and compression co-exist at every point in the tissue due to Poisson effects (Lai et al., 1995). However, tension drives pathology more than compression does because neurites are long slender structures (Bandak, 1995). Such structures break in tension but collapse in compression without damage. Recent mathematical simulations of clinical head injury events have employed MPS thresholds for damage of 0.29 (Hajiaghamemar et al., 2020), 0.15 (Sanchez et al., 2019) and 0.25 (Gabler et al., 2016). These thresholds concur with our results, which showed increases in injury phenotypes across MPS ranges spanning these thresholds (Figs 4, 5).

Human cortical spheroids reproduced injury phenotypes observed in primary rodent tissue or cancer cell lines after *in vitro* neurotrauma. Trauma reduces cell viability in these models (Ellis et al., 1995; LaPlaca et al., 2005; Morrison et al., 2006) as it does in this model (Fig. 4B,C). Mitochondria malfunction in other *in vitro* neurotrauma models (Wang et al., 2014; Ellis et al., 1995), as they do in this model (Fig. 4D). Capturing mitochondrial dysfunction *in vitro* is important for drug discovery. Cyclosporin improved TBI outcomes in a pig model by protecting mitochondria (Karlsson et al., 2018). The only medium-throughput screening study focused on TBI that we know of produced a hit that protected mitochondria (Lopez-Garcia et al., 2018). *In vitro* stretch injury reduced intraneuronal NF-L levels in primary rat neurons (Jackson et al., 2018), consistent with our finding of increased release of NF-L after trauma (Fig. 4D). This result raises the exciting possibility of using clinically validated TBI biomarkers as outcomes in a drug discovery screen employing traumatized human cortical spheroids. Spontaneous synchronous activity declined at moderate injury levels that did not cause pronounced cell death (Fig. 5). This pattern is consistent with previous literature reporting that mild trauma alters synaptic receptor activity without cell death in primary rodent neurons (Goforth et al., 2011). Trauma briefly boosts and then depresses N-methyl-D-aspartate receptor function *in vitro* (Goforth et al., 2011) and *in vivo* (Biegon et al., 2004), and also boosts GABA function (Kao et al., 2004). These synaptic changes reduce long-range connectivity and synchrony in two-dimensional rodent neuron monolayers (Patel et al., 2012). Similar changes occur in rodent organotypic slice cultures after stretch injury (Kang and Morrison, 2015). Therefore, changes in synaptic receptors are the most likely mechanism for the loss of spontaneous synchronous activity in this study.

Although this model has biomechanical injury thresholds and pathological phenotypes consistent with previous literature as discussed above, it also has unique strengths. Compared to two-dimensional monolayer cultures, three-dimensional spheroidal cultures have the advantage of reproducing three-dimensional mechanical strain fields. This advantage is important in neurotrauma research because compression, tension and shear strain are not orthogonal biomechanical quantities. They are coupled together by Poisson effects (Lai et al., 1995), and this phenomenon can only be reproduced in a three-dimensional structure. Human cell cultures have advantages over primary rodent cells because they eliminate species differences that have defeated many efforts to translate positive preclinical studies into clinical trial success for TBI (Stein, 2015). Also, patient-specific and isogenic human *in vitro* models can be used to model patient heterogeneity and the role of individual genetic variants in disease, respectively (Warren and Cowan, 2018; Kim et al., 2014). These phenomena are extremely important with TBI because patient heterogeneity is an important factor in the repeated failure of clinical trials (Gardner et al., 2019; Maas et al., 2010) and several common genetic variants have been shown to drive some of this heterogeneity (Barbey et al., 2014; Lipsky et al., 2005; Yue et al., 2015). Finally, self-organized spheroidal three-dimensional cultures have advantages over three-dimensional cast hydrogel cultures because they are more electrophysiologically active. The higher cell density of self-organized cultures allows more synapses to form and these synapses lead to robust spontaneous synchronous activity. This advantage allows this model to report electrophysiological phenotypes in addition to cell viability phenotypes. Taking these points together, this model is distinguished from previous models by the three-dimensional character of the mechanical insult, the genetic tractability, the human origin of the cells and the functional outcomes. However, the unique nature of the model creates limitations as well as opportunities. At present, human brain organoids exist in an embryonic state of maturity and therefore may not match the injury response of the adult or aged brain. For example, the neuroplasticity of these cultures may be greater than a more mature brain. Nevertheless, brain organoids can reproduce pathology of adult disease. For example, cerebral organoids formed aggregates of amyloid-β and phosphorylated tau proteins in an Alzheimer's disease drug screen (Park et al., 2021). Additionally, the prospects for mature brain organoids in the near future are bright. Cortical organoids were recently shown to reach postnatal levels of maturity in long-term culture (Gordon et al., 2021).

In summary, we found that human cortical spheroids provide a biofidelic multifaceted TBI phenotype, including changes in viability, mitochondrial function, electrophysiology and biomarker release. This phenotype agrees with much of what has been learned from previous *in vitro* models of TBI employing cancer cell lines or primary rodent tissue. This platform can bring the unique advantages of hiPSC-derived cells to bear on the vexing problem of TBI. Target-based drug discovery is difficult in TBI because the pathology is multimodal. Phenotypic drug discovery is difficult because established preclinical models are low throughput. Outcomes vary widely across patients for poorly understood reasons, making it hard to achieve adequate statistical power in clinical trials. Three-dimensional human *in vitro* models can be scaled up for high-content phenotypic drug discovery and genetically matched to patients to inform clinical trial stratification. Therefore, this platform has the potential to open up a new horizon in the search for personalized effective treatments for TBI.

## MATERIALS AND METHODS

### Cortical spheroid culture

StemoniX supplied the cortical spheroids used in this study under the brand name microBrain 3D (BSARX-AA-0384). These spheroids were selected for their geometric consistency and robust spontaneous activity. They were generated as follows: neural progenitor cells (NPCs) were isolated from neural rosettes derived from hiPSCs, according to a previously published protocol (Marchetto et al., 2010). These NPCs were cultured in a spheroidal format and matured into neurons and astrocytes using a published protocol that promotes activity (Gunhanlar et al., 2018). The amount of necrosis at the core was small under these culture conditions (Fig. S3, core necrosis is a near-universal feature in cultures of this type). Published data confirm that this technique produces spheroids with mature astrocytes (indicated by Aquaporin 4 and glial fibrillary acidic protein staining) and mature neurons (indicated by MAP2 and synapsin I staining) (Sirenko et al., 2019). These cultures were maintained using a NeuralX Cortical Neuron media kit (StemoniX, NXCNM-AA-0250) with 1× penicillin-streptomycin (GE Healthcare Life Sciences). Half medium changes were performed three times on the day the spheroids were delivered and three times a week thereafter. Spontaneous electrophysiological activity emerged after 8 weeks in culture and persisted up until ∼20 weeks in culture. As electrophysiological outcomes were important in this study, experiments were performed with spheroids that had been in culture for 10-18 weeks. All outcomes were normalized to on-plate controls to eliminate batch-dependent and age-dependent effects.

### Injury device and procedure

The custom-built injury device consisted of a flat stationary plate platform mounted above a vertically translating stage (Fig. 2A,B). The stage supported an aluminum block with a hole pattern matching the well locations in a standard 96-well plate. A LA43-67-000A voice coil (BEI Kimco) moved the stage vertically. A Xenus XTL-230-40 servo drive (Copley Controls) controlled the voice coil using closed loop feedback from a T1031-30A linear quadrature encoder (Renishaw PLC) that measured the stage position. To injure spheroids, we first used wide bore pipette tips to transfer them from the 96- or 384-well plates in which they were routinely maintained into a custom-built 96-well plate with a stretchable transparent PDMS well bottom (Sherman et al., 2016; Phillips et al., 2018), one spheroid per well. A custom-built vacuum apparatus was applied to the bottom of the plate to draw the flexible well bottoms into a concave shape temporarily so the spheroids would move to the center of the well. Then, the vacuum was released gently to restore the flat shape of the membrane while keeping the spheroids centered (Fig. S2). Cylindrical flat-ended indenting posts were mounted in the block on the indenter stage. We built a lid that fitted over the 96-well plate. This lid consisted of an aluminum block with holes collocated with the 16 wells in the middle of the 96-well plate (Fig. 2C). We attached collars to the ends of stainless steel posts with set screws and dropped them into the holes in the lid. The collars rested on the lid so the bottom of the post hung above the well bottom. We positioned the collars precisely using a micrometer so the distance between the bottom face of the post and the well bottom would equal the average height of the spheroids in that experiment (Fig. 2D; Fig. S2I). We placed the lid on the plate in a cell culture hood and moved it to the injury device (note that the lid and posts were sterilized in an autoclave beforehand). At wells designated for sham injury, we omitted the indenters and replaced the drop-in posts with short aluminum plugs that served merely to maintain the sterility of the cell culture medium. We raised the stage slowly until the indenters contacted the bottom of the stretchable plates so that the spheroid rested on a flexible PDMS membrane between two flat rigid surfaces. We then injured the spheroids by applying a vertical displacement pulse with an amplitude of several hundred microns (Fig. 2E). The duration of the pulse was 30 ms for all experiments except those addressing strain rate, in which longer pulse durations were also used.

### Finite element model of injury

We modeled spheroids in ABAQUS as spheres with a radius of 300 µm loaded by rigid surfaces with a coefficient of friction of 0.5. A two-dimensional axisymmetric quadrant representation took advantage of the axial and horizontal symmetry of the problem to reduce computational time. We modeled the tissue with a fully incompressible neo-Hookean hyperelastic constitutive law. Coefficients for the material were fitted to experimental stress-strain data from high-speed uniaxial tension and compression of excised human grey matter (Jin et al., 2013) (note that the strain field is insensitive to the stiffness because it is driven by an applied displacement, not an applied traction). As explained in the Results, the outermost region of the spheroid was partitioned so that the strain distribution in the tissue visible to the confocal microscope (Fig. 3A) could be considered independently of the whole spheroid (Fig. 3B). We used the Image Processing Toolbox (Matlab) to segment images of spheroids labeled with the calcium reporter Calcium 6 and the cell viability indicator Calcein AM. Adaptive meshing tools in the software provided a convergent efficient triangular mesh for the partitioned geometry (Fig. 3B). A displacement boundary condition on the rigid surface compressed the mesh. We simulated 14 compression magnitudes, ranging from 5% to 70% in 5% increments. We computed volume-averaged MPS for the visible domain (MPS_visible_) and whole spheroid (MPS_whole_) from the simulation output, and interpolated between compression ratios when necessary using a fourth order polynomial (Fig. 3D).

### Fluorescent microscopy

To measure electrophysiological activity, we stained spheroids with the fluorescent calcium indicator calcium 6 (Molecular Devices) according to the manufacturer's instructions. Briefly, we reconstituted the dye in Hank's balanced salt solution to a 2× stock concentration, replaced half the culture medium with it and continued incubation at 37°C and 5% CO_2_ for 2 h before starting imaging. We imaged each spheroid at 1 Hz for 150 s using a Nikon Plan Fluor 10× DIC, 0.3 NA objective and a Nikon C2 Plus single-photon confocal on a Nikon Ti Eclipse inverted microscope with FITC filter and laser settings. The cultures were maintained at 37°C and 5% CO_2_ using a Tokai Hit stage top incubator during imaging, which took no more than 1 h. We segmented the images using Image Processing Toolbox and calculated the history of the average intensity in the spheroid area. We used the findpeaks function (Matlab) to identify peaks in this history (Fig. 5A). We visually inspected the output and, when necessary, adjusted function parameters and repeated the process to eliminate errors.

To measure cell viability and mitochondrial membrane potential, we stained spheroids with Hoechst 33342 (MilliporeSigma), Calcein AM (Invitrogen) and TMRM (MilliporeSigma) at 10 µg/ml, 1 µM and 100 nM, respectively, for 2 h at 37°C and 5% CO_2_ starting 22 h after injury. We generated three-dimensional stacked images of the spheroids using a Nikon Ti Eclipse inverted microscope (Nikon Plan Fluor 10× DIC, 0.3 NA) and a Nikon C2 Plus single-photon confocal system with DAPI, FITC and TRITC filter and laser settings. We acquired 31 slices with a step size of 10 µm to image the visible portion of the spheroid. Then, we reduced the image stack to a maximum intensity projection in ImageJ, segmented on the DAPI channel in Matlab and averaged the intensity in the spheroid area in the Calcein AM and TMRM channels.

### Measurement of LDH

We collected 50 µl of spheroid culture medium before injury and another 50 µl 3 h after injury. We measured LDH concentration in these samples using an CytoTox 96 Assay kit (Promega) according to the manufacturers' instructions. Briefly, we added 50 µl of CytoTox 96 reagent to the sample for 30 min at room temperature, then added 50 µl of stop solution and measured the absorbance in each well using a Synergy HTX plate reader (BioTek Instruments) using the 492 nm laser line. We subtracted baseline values measured in samples of naïve cell culture medium and then computed the change in LDH concentration between the before and after injury samples for each spheroid.

### Measurement of NF-L

We used a human NF-L ELISA kit from Novus Biologicals to measure NF-L. We combined two undiluted 50-µl samples that received the same treatment (injury or sham) in order to load 100 µl/reaction in the provided 96-well ELISA plate. The reference standards (ran in duplicate) and experimental samples were allowed to incubate overnight for 16 h at 4°C. The samples and standards were aspirated, and 100 µl of biotinylated detection antibody was added and incubated for 1 h at 37°C, followed by aspiration and five washes with washing buffer. Next, 100 µl of horseradish peroxidase conjugate was added and incubated for 30 min at 37°C, followed by aspiration and five washes. Finally, 90 µl of substrate reagent was added for 15 min at 37°C, followed by 50 µl of stop solution, and the absorbance in each well was measured on a Synergy HTX plate reader (BioTek Instruments Inc.) using optical density at 450 nm and wavelength correction at 540 nm. Background noise was corrected for by subtracting the average absorbance value of the zero standard (i.e. sample diluent only). The concentration of NF-L was calculated from the standard curve.

### Statistical methods

Hypotheses about correlations between injury phenotypes and the magnitude or rate of mechanical deformation were tested using linear regression. The reported *P*-values quantify the probability that the true slope of the regression is zero (i.e. that the hypothesized correlation does not exist).

## Supplementary Material

Supplementary information
