# Supplementary material for: Biofidelic dynamic compression of human cortical spheroids reproduces neurotrauma phenotypes
Source: Dis Model Mech. 2021 Dec 22;14(12):dmm048916. doi: 10.1242/dmm.048916 (PMC8713991; doi:10.1242/dmm.048916)
Supplement: Supplementary information [file dmm-14-048916-s1.pdf]

Supplementary Materials and Methods

Spheroid Metrology

The degree of injury applied to a spheroid depends on the spheroid's height. Since it is difficult to measure the height of three-dimensional cultures in a high-throughput manner, we tested the hypothesis that spheroid height is similar to spheroid width (which is a more convenient measurement using conventional microscopy methods). We constructed an imaging apparatus that allows both the top and side of a spheroid to be imaged on an inverted microscope (Fig. 1A). The apparatus consisted of a small optical prism (Edmund Optics) that was mounted to the bottom of a 60mm petri dish using vacuum grease. This design followed the imaging apparatus described by Elkin et al. (Elkin et al., 2010), where slices of rat brain tissue were imaged from the bottom and side to estimate volume. Our imaging apparatus incorporated a small acrylic corral mounted adjacent to the prism, which confined the spheroid within a small region of the dish. A waterproofed LED (Cree, Inc) was mounted to the bottom of the dish such that it transmitted light through the spheroid corral toward the optical prism. This apparatus, when mounted to the stage of an inverted microscope, allowed for transmitted white-light microscopy in both the horizontal and vertical planes. We captured side and top views of 24 spheroids using a Nikon Ti Eclipse inverted microscope (Nikon Inc.; Nikon Plan Fluor 10x DIC, 0.3 NA) and an Andor Neo brightfield camera (Andor Technology), as shown in Figure 1B, C. We manually segmented each captured image in Matlab to fit an unconstrained ellipse around the spheroid. The segmentation algorithm recorded the major axis, minor axis, and projected lengths along the global X- and Y- axes for each view. The minor axis in the view from below was correlated against the Y-axis projection in the view from the side, which was selected as the best estimate of spheroid height.

Prior to each injury assay, we captured brightfield images of the spheroids using an inverted microscope (Nikon Plan Fluor 10x DIC, 0.3 NA) and segmented them using Matlab. We recorded the minor axis of each segmented image and used the average minor axis of the spheroid batch to estimate the average height of the spheroid batch. Table S1 lists the average minor axis measurement and standard deviation for eight batches of spheroids prior to injury.

Injury Device Metrology

We broke the challenge of maximizing the consistency of the compressive insult down into five tasks: (1) ensure that the stage and the platform on the injury device (Fig. 2) are parallel, (2) ensure that the faces of the indenters form a flat surface, (3) ensure that the faces of the drop-in posts are flat, (4) define the initial position for the stage to contact the plate without deforming the spheroids, and (5) compress the spheroids with a consistent displacement pulse. These components are addressed in turn in the following paragraphs.

(1) The injury device stage is supported on 3 pillars using spherical washers that allow a small amount of rotation. Two of these pillars can be independently lengthened and shortened to adjust both the roll and pitch of the stage. We adjusted these pillars until the distances between the stage support points and the plane of the plate platform were consistent to within ±0.0005", indicating parallelism between the indenter stage and the plate platform. Following alignment of the stage, we clamped the two adjustable pillars in place using shaft collars to ensure that the parallelism of the device was permanently fixed.

(2) We manufactured the indenters and drop-in posts using 316 stainless steel, which was chosen because it can be surface-ground with high precision. We mounted the indentation posts in the indenter post array (Fig. 2), and ground the entire assembly to ensure that the posts were flat and coplanar. Following surface grinding, we verified the coplanarity of the indentation post array using a dial indicator (Yuasa International). The

variance in height between each post in the array was beyond the indicator's detection limit of ±0.0005 inches, meaning that the post array was coplanar to at least ±12.7µm.

(3) We surface ground the ends of the drop-in posts individually, and verified their flatness using a ContourGT white-light interferometer (Bruker). A representative contour map of a drop-in post is shown in Figure S1A. The average root-mean-squared surface roughness across the drop-in posts was ± 903.39 nm.

(4) In order to identify the indenter stage position that created contact between the indenters and the bottom of the 96-well plate, we placed the custom hanging post lid on a bottomless Nunc plate, and inserted four drop-in posts into holes in the lid. We positioned the four drop-in posts such that their bottom ends would be coplanar with the bottom surface of the 96-well plate. Next, we placed the plate on the injury device's platform and positioned a dial indicator on top of one of the drop-in posts. We incrementally moved the stage upwards until the indenter contacted the drop-in post, causing the needle of the dial indicator to deflect, and recorded the stage position. We repeated his process nine times per well for eight of the central wells in the 96-well plate. Contact positions for the eight post array used in injury experiments are plotted in Figure S1B. The color scale represents the indenter stage position, in absolute encoder units, where contact between the posts was identified. The average contact position was 9555.5 ± 6.35 µm. The range between the highest contact point (Well E07) and the lowest contact point (Well F05) was 20.60µm.

(5) We verified consistency in displacement amplitude by recording displacement traces during trial runs of the device using the T1031-30A linear quadrature encoder (Renishaw PLC). We loaded a flexible PDMS plate with 150 µL of phosphate buffered saline solution (Gibco) per well, fitted it with the drop-in post assembly, and placed it on the plate platform of the injury device. We executed a total of eight displacement pulses, with nominal amplitudes ranging from 100µm to 450µm in increments of 50µm. The target duration of each pulse was 30ms, regardless of pulse amplitude. We repeated each nominal displacement pulse 10 times, for a total of 80 trials. We randomized the sequence of displacement amplitudes, so any effect of repeated indentation on the PDMS plate was spread evenly across the experiment. During each trial, we sampled the position of the encoder at 10kHz using a Compact DAQ system, and plotted it using LABVIEW (National Instruments). Typical displacement traces for a nominal displacement of 250µm are shown in Figure S1C. The amplitude and duration of each pulse was consistent between all 10 trials. Regardless of the amplitude of displacement, the injury device was able to update its velocity such that the duration of the injury pulse was consistently held at the nominal target of 30ms, as summarized in Table S2. The correlation between nominal displacement and actual displacement is shown in Figure 2D. The slope of this regression is 1.0895, indicating that there is a slight offset between the nominal displacement that the user inputs into the system and actual displacement achieved by the device during injury. However, the R^2 value of 0.9995 indicates that this offset is consistent across a wide range of displacement amplitudes, and can thus be accounted for while designing injury experiments.

Fluorescent Electrophysiology

We measured spontaneous electrical activity at varying levels of mechanical strain. During analysis, we normalized data from injured spheroids to sham data from spheroids on the same plate. We plotted the normalized calcium peak count and peak intensity data as a function of MPS, which we calculated on an individual spheroid basis using the height of the spheroid prior to injury, and the actual amplitude of displacement the spheroid experienced during injury. We fit a

sigmoidal regression curve to the peak count and peak intensity datasets. The fitted sigmoid equation follows the form:

$$y = d - \frac{d - a}{1 + \left(\frac{x}{c}\right)^{b}}$$

where *d*, *a*, *c*, and *b* represent the sigmoid's maximum, minimum, inflection point, and decay rate, respectively. We estimated the sigmoid parameters using the Levenberg–Marquardt fitting algorithm in Matlab's Curve Fitting Toolbox. Initial guesses for *d*, *a*, *c*, and *b* were 1.0, 0.0, 25.0, and -1, respectively. Additionally, the parameter *a* representing the floor of the sigmoid was bounded to be greater than or equal to zero. Fitted sigmoids for peak count and peak intensity are shown in Figures 5C and 5D, respectively. Regression statistics for these two curves are summarized in Table 3.

Live/dead imaging

To visualize the proportions of live and dead cells at baseline, spheroids were stained with 20 µM Hoescht 33342 (Life Technologies), 2µM Calcein-AM (Thermo Fisher Scientific), and 1µM Propidium Iodide (Thermo Fisher Scientific) 1 hour prior to injury. Images were collected at 10X magnification using a Leica DMi8 inverted light microscope. Images shown were acquired using the DAPI and Texas Red filter settings.

In these images, Hoechst labels all nuclei, but propidium iodide only labels the nuclei of dead cells because it cannot cross intact plasma membranes. The pattern of staining clearly reveals a necrotic core inside each of the spheroids examined. This phenomenon is almost universal in neural organoid cultures and arises from the absence of vasculature. In the human brain, a fine network of capillaries delivers oxygen and glucose to within tens of microns of each cell. There is no analogous network in an organoid so diffusion is the only process delivering oxygen and glucose from the media around the culture to the interior. Diffusive transport is insufficient to support cells at the center of the organoids so a necrotic core forms. Fortunately, in these spheroids, the necrotic core is small relative to the total volume of the organoid (see Fig. S3). It is not surprising that the necrotic core is small because the overall spheroid diameter is small relative to other commonly used protocols. The small size of the spheroids facilitates diffusion of nutrients to the interior.

High magnification imaging of Calcein AM, Hoechst and TMRM

An Olympus IX83 confocal microscope was used to acquire 3D image stacks of spheroids labeled with Calcein AM, Hoechst 33342 and tetramethylrhodamine (TMRM). The staining protocol was as described in the Methods section of the manuscript. Images were collected with a 20X 0.75NA objective and experimental samples were maintained in a humidified Tokai Hit stage top incubator at 37 °C and 5% CO_2 during imaging. Each slice was 2048 x 2048 pixels with a 0.124 µm pixel size and a 2 µm slice interval. Each stack took 30 minutes to acquire so this scheme would have been impractical as a way to collect complete multi-category data sets for hypothesis testing. However, it was appropriate for visualizing the distribution of staining at the cellular length scale that generated the staining observed at the whole spheroid scale in Fig. 4. 3D image stacks were reduced to a single frame by maximum intensity projection.

Calcein AM uniformly stained the cells in the spheroid (see Fig. S4). The cells are densely packed with very little extracellular matrix separating them so it is not possible to distinguish single cell outlines from the environment. However, many neurites and a handful of soma are visible. This observation is consistent with prior reports that neurites tend to dominate over soma

Disease Models & Mechanisms • Supplementary information

in the periphery of spheroidal human-origin neural cell cultures (Renner et al., 2020). The Hoechst 33342 channel reveals that the majority of nuclei are large, moderately stained and ellipsoidal. The TMRM channel reveals punctate staining of small round sub-cellular structures. In some instances, these combine into long slender chains. This distribution is expected for mitochondrial staining of highly polarized neurons with long slender neurites (Varkuti et al., 2020).

High magnification imaging of calcium dynamics

An Olympus IX83 confocal microscope was used to acquire time series images of spheroids stained with Calcium 6. The Calcium 6 staining protocol was as described in the Method section of the manuscript. Images were collected with a 20X 0.75NA objective and experimental samples were maintained in a humidified Tokai Hit stage top incubator at 37 °C and 5% CO_2 during imaging. 334 x 512 pixel images with a 0.497 µm pixel size were acquired at a rate of 1 Hz for 150 seconds. The synchronicity of the calcium dynamics within the culture were inspected using a method presented by Renner et al. (Renner et al., 2020) that involved comparing the intensity history of the calcium fluorescence signal for the whole culture to the history for individual cells. The cell density in the cultures was too high to permit segmentation of whole cells. However, individual neurites were visible that were clearly specific to a single cell. ROIs were drawn around these neurites in Fiji and the calcium intensity history within each one was calculated. These intensity histories were plotted with Matlab alongside the global intensity history (i.e., the intensity history of the whole field of view) (see Fig. S5).

There were peaks in neurite-specific ROIs at the same time as the peaks in the global intensity history (see Fig. S5). These results indicate that the calcium dynamics of the culture were highly synchronous. This observation is consistent with previous reports of highly synchronous electrophysiological activity in spheroids of human-origin neural cells (Renner et al., 2020).

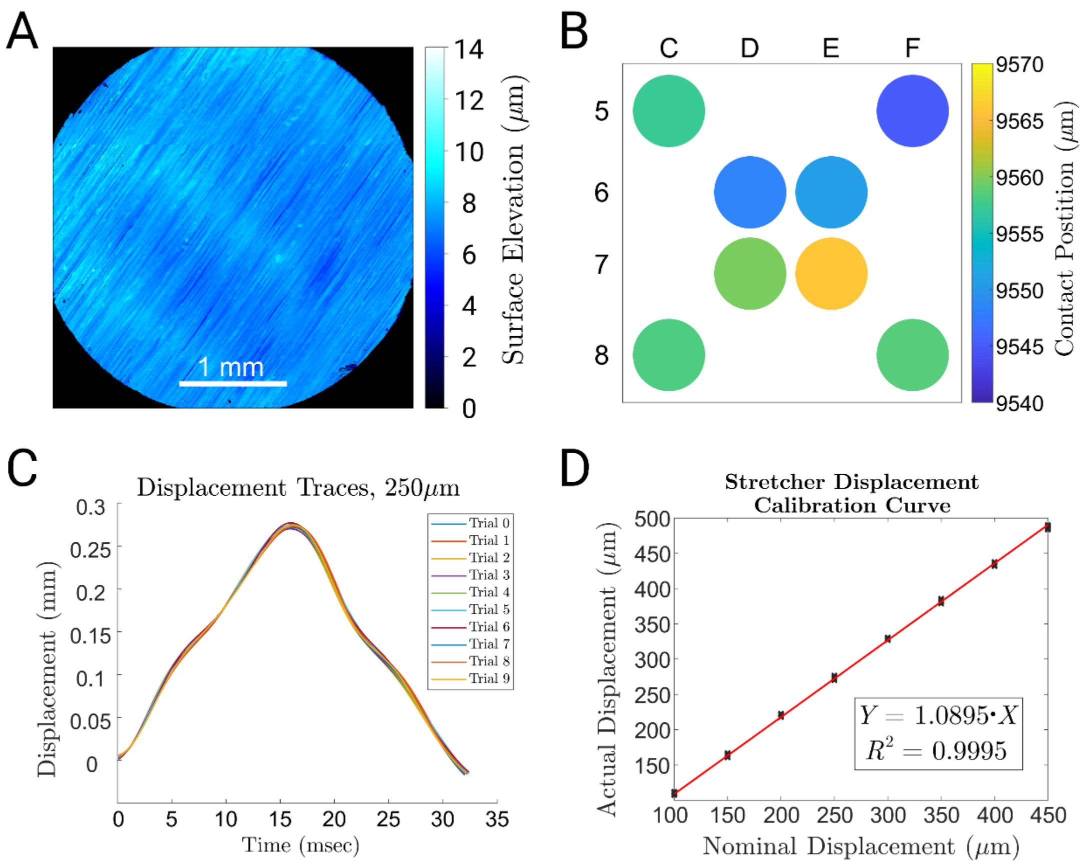

Fig. S1. Characterization of the device for sub-millimeter spheroid injury. (A) White-light interferometry surface map of a typical drop-in post following surface grinding. (B) Heat map of inter-well variation in the indenter stage position at which contact occurs between the indenter post and the corresponding drop-in post. (C) Displacement traces collected from repeated trials with a nominal amplitude of 250µm (n = 10). (D) Regression of nominal displacement amplitude to actual displacement amplitude.

Disease Models & Mechanisms • Supplementary information

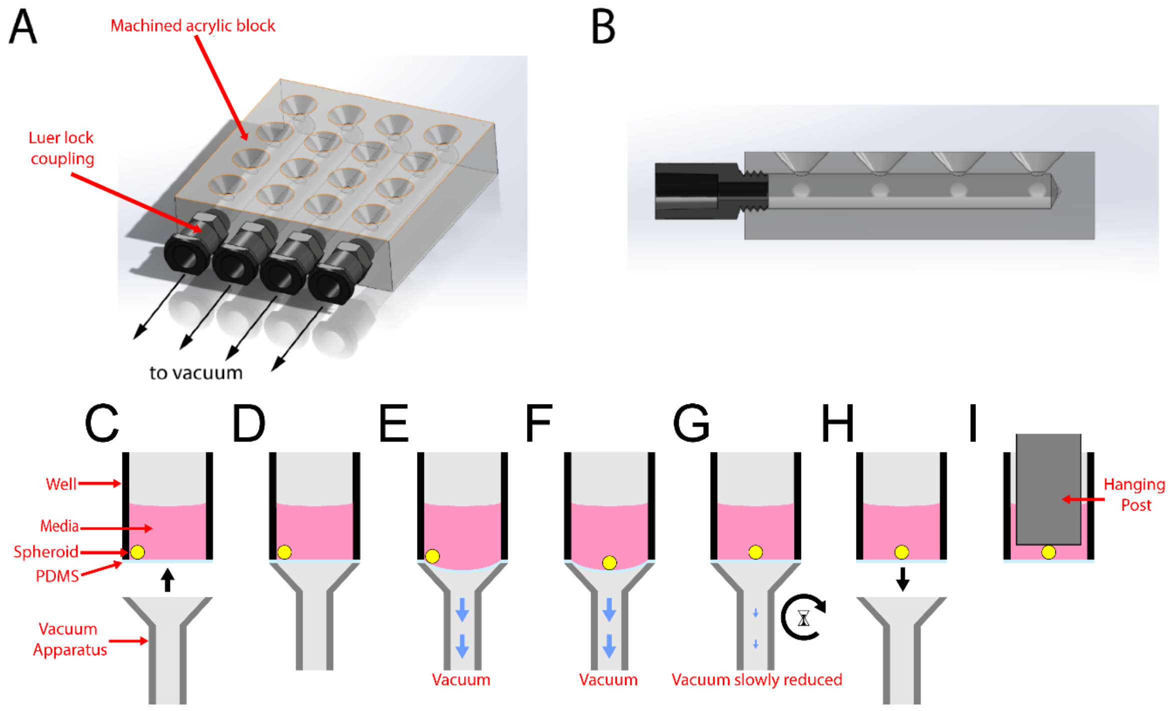

Fig. S2. Apparatus for centering spheroids in stretchable plates. (A) Isometric view. (B) Cross sectional view. (C-D) The block was placed against the bottom of the plate, the block's holes aligned with the well bottoms. (E) When suction was applied, the flat PDMS membranes were temporarily drawn into a conical shape. (F) Gravity moved each spheroid to the center of its well. (G) Suction was then released gradually, returning the membranes to a flat shape without displacing the spheroids from the well centers. (H) Block removed. (I) Finally, a hanging post was suspended from the plate lid so that the bottom face contacted the spheroid.

Disease Models & Mechanisms • Supplementary information

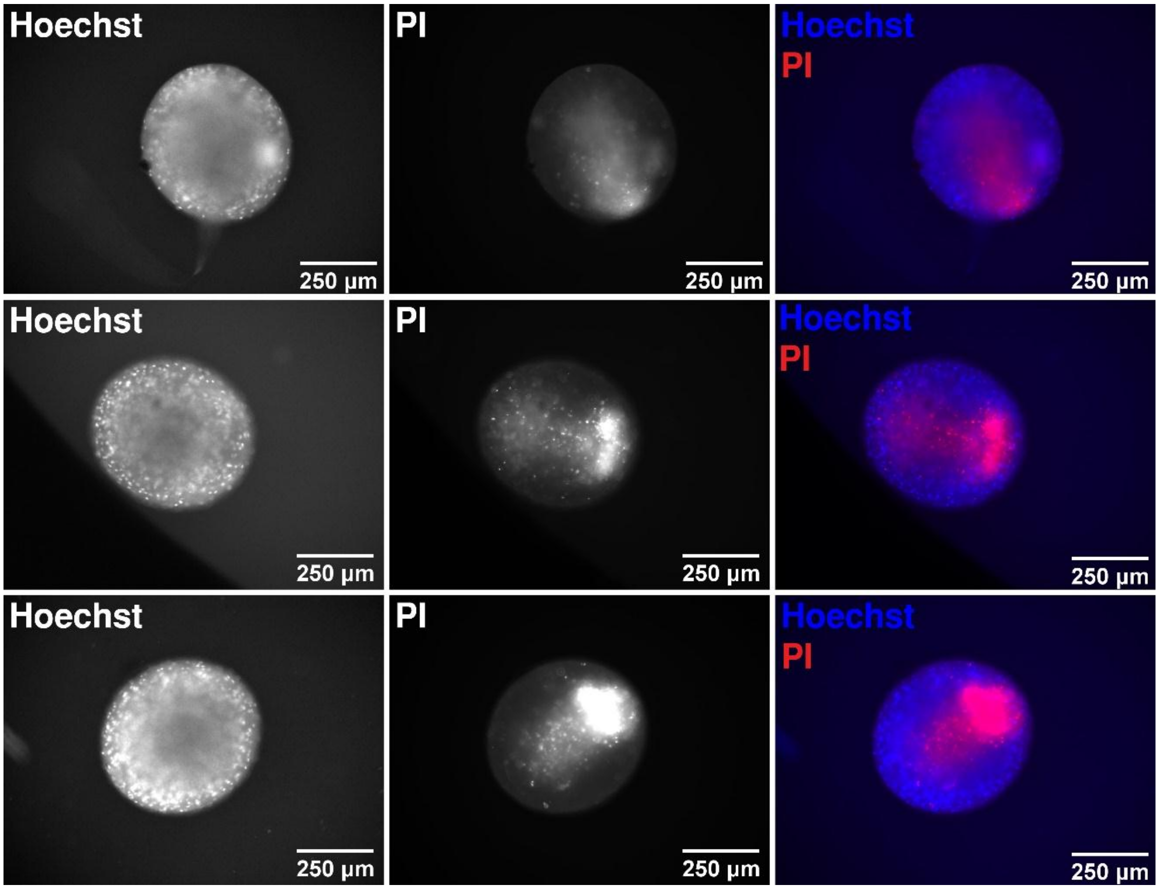

Fig. S3. 3 spheroids (a different example in each row) stained for all nuclei (Hoechst) and nuclei in dead cells (propidium iodide, PI).

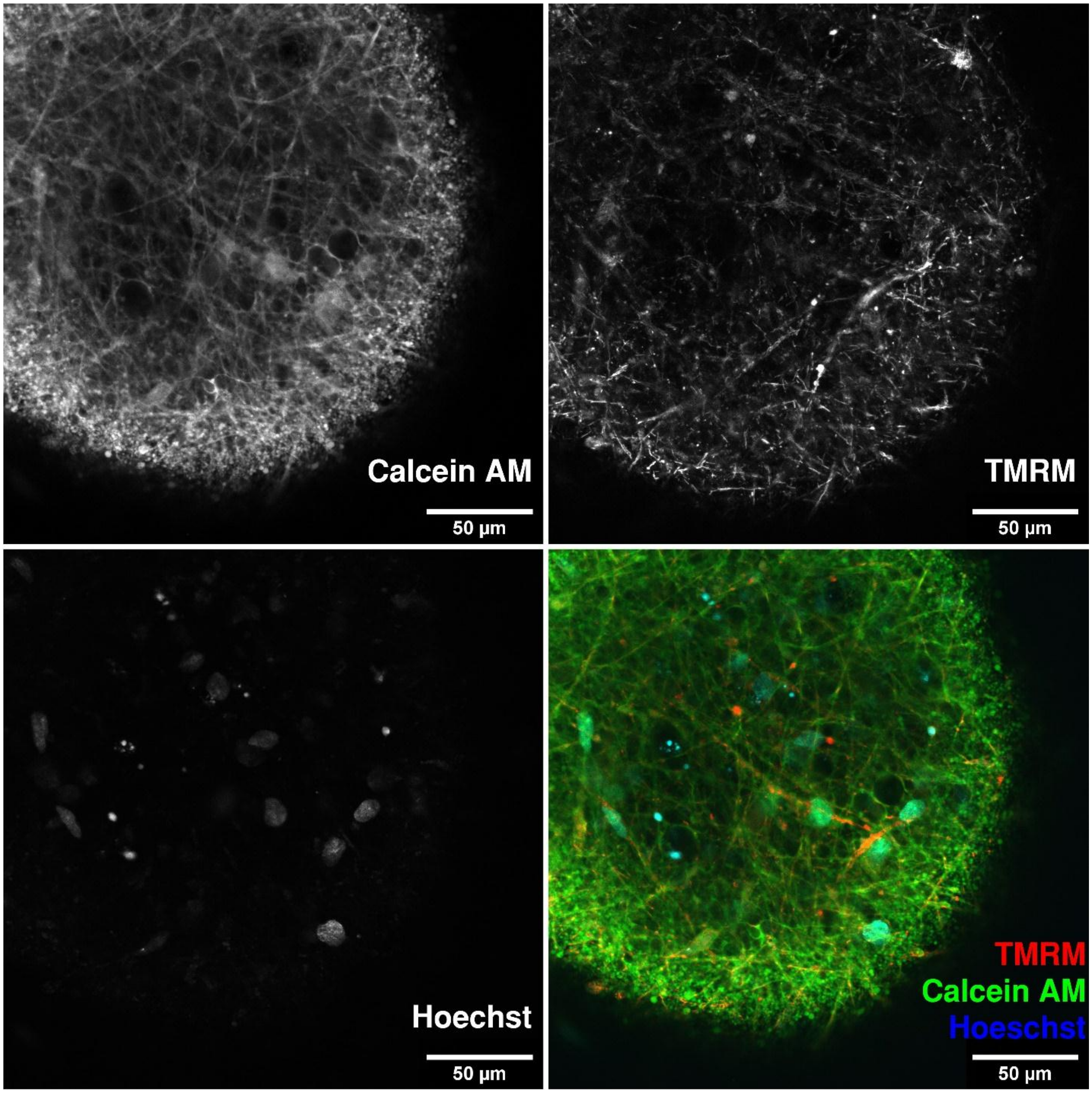

Fig. S4. High power images of spheroids stained with Calcein AM, Hoechst 33342, and TMRM. This data set is typical of six repetitions of this experiment.

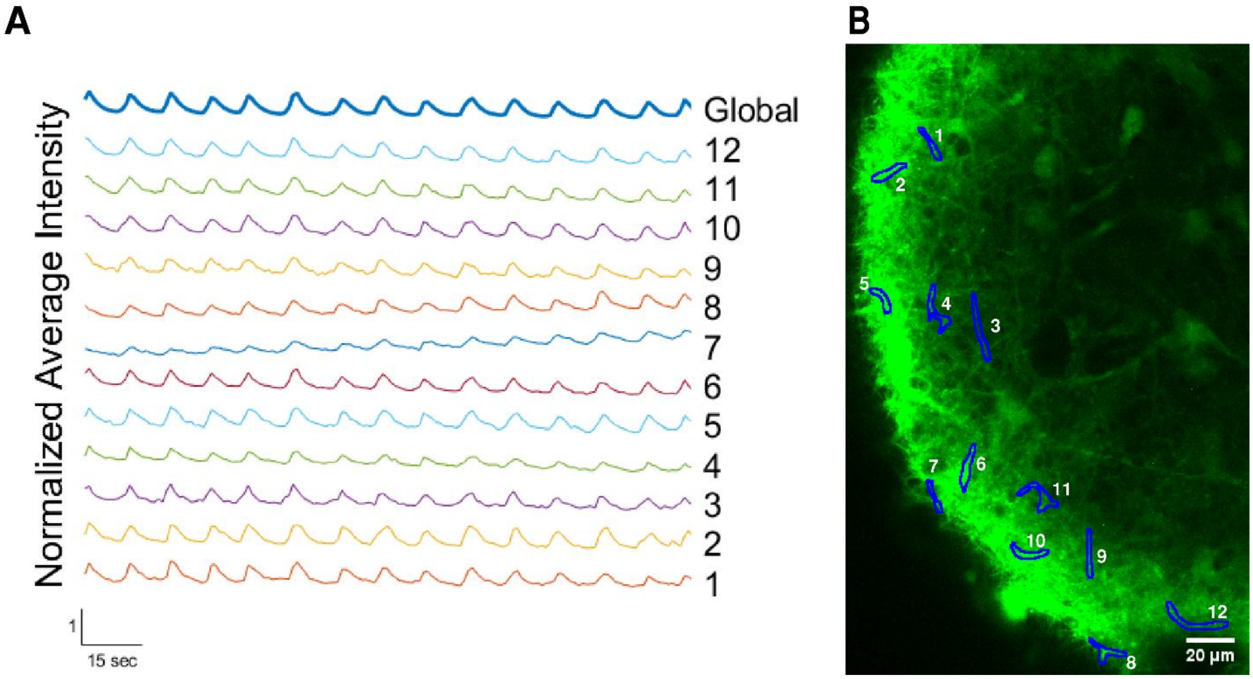

Fig. S5. Synchronous calcium dynamics. (A) Calcium intensity histories for 12 regions of interest (ROIs) specific to individual neurites that were selected at random, along with the global average of the calcium intensity for the whole image. (B) The ROIs corresponding to the traces displayed in panel A. These results are typical of 6 repetitions of this experiment.

Disease Models & Mechanisms • Supplementary information

Table S1. Minor axis measurements for eight batches of spheroids prior to injury assay.

Spheroid Age (Weeks.Days)	*N*	Mean Minor Axis (μm)	Std Dev Minor Axis (μm)
11.3	95	630.17	16.31
17.2	29	488.65	18.86
15.3	94	554.66	19.04
10.6	93	618.02	10.37
12.4	124	591.40	10.09
11.6	96	528.00	12.00
12.5	96	522.80	12.60
11.6	96	579.42	16.37

Table S2. Summary of injury duration statistics for trial (n = 10) of varying amplitude.

Nominal Displacement (μm)	Pulse Width (ms), Mean ± Std Dev
100	29.62 ± 0.42
150	29.59 ± 0.32
200	30.31 ± 0.21
250	30.07 ± 0.16
300	30.39 ± 0.17
350	30.74 ± 0.16
400	30.55 ± 0.21
450	30.94 ± 0.13

Table S3. Regression statistics for fitted sigmoid curves describing electrophysiological responses to mechanical strain. *The floor parameter *a* is bounded such that it is greater than or equal to zero; all other parameters remain unbounded.

Data Fitted	Sigmoid Parameters				df_{Err}	MS_{Err}	R^2
	d	a*	c	b			
Peak Count	1.105	3.82E-6	25.37	-7.05	107	0.313	0.675
Peak Intensity	0.9972	6.21E-5	28.06	-17.06	107	0.306	0.698

SI References

ELKIN, B. S., SHAIK, M. A. & MORRISON, B., 3RD 2010. Fixed negative charge and the Donnan effect: a description of the driving forces associated with brain tissue swelling and oedema. *Philos Trans A Math Phys Eng Sci,* 368**,** 585-603.

RENNER, H., GRABOS, M., BECKER, K. J., KAGERMEIER, T. E., WU, J., OTTO, M., PEISCHARD, S., ZEUSCHNER, D., TSYTSYURA, Y., DISSE, P., KLINGAUF, J., LEIDEL, S. A., SEEBOHM, G., SCHÖLER, H. R. & BRUDER, J. M. 2020. A fully automated high-throughput workflow for 3D-based chemical screening in human midbrain organoids. *Elife,* 9.

VARKUTI, B. H., KEPIRO, M., LIU, Z., VICK, K., AVCHALUMOV, Y., PACIFICO, R., MACMULLEN, C. M., KAMENECKA, T. M., PUTHANVEETTIL, S. V. & DAVIS, R. L. 2020. Neuron-based high-content assay and screen for CNS active mitotherapeutics. *Sci Adv,* 6**,** eaaw8702.
